# Evaluation of a low-cost, low-power syringe pump to deliver magnesium sulfate intravenously to pre-eclamptic women in a Malawian referral hospital

**DOI:** 10.1186/s12884-017-1382-9

**Published:** 2017-06-19

**Authors:** Erica Skerrett, Edward Kommwa, Kelley Maynard, Alexa Juarez, Ronald Mataya, Rebecca Richards-Kortum, Z. Maria Oden

**Affiliations:** 1 Rice 360: Institute for Global Health Technologies, Rice University, Houston, TX USA; 2 Department of Obstetrics and Gynecology, Queen Elizabeth Central Hospital, Blantyre, Malawi; 3 Department of Obstetrics and Gynecology, University of Malawi College of Medicine, Blantyre, Malawi; 4 School of Public Health, Loma Linda University, Loma Linda, California USA; 5 Department of Bioengineering, Rice University, Houston, TX USA

**Keywords:** Global Health, Infusion pump, Low-resource settings, Magnesium sulfate, Malawi, Maternal Health, Pre-eclampsia, Syringe pump

## Abstract

**Background:**

Magnesium sulfate is an affordable and effective treatment for pre-eclampsia and eclampsia. In settings where infusion pumps are not available to regulate the flow rate of intravenous delivery, healthcare providers must administer magnesium sulfate (MgSO_4_) via time-consuming and painful, large-volume intramuscular injections. As an alternative to costly commercially available syringe pumps, we developed AutoSyp, an accurate, low-cost, and low-powered syringe pump designed to meet the needs and constraints these low-resource settings. This paper describes results of a pilot study to evaluate the feasibility of using AutoSyp to administer MgSO_4_ intravenously to women suffering from pre-eclampsia at a referral hospital in Blantyre, Malawi.

**Methods:**

AutoSyp was programmed to deliver MgSO_4_ following the Zuspan regimen to pregnant and post-partum women suffering from pre-eclampsia at Queen Elizabeth Central Hospital in Blatnyre, Malawi. Given the selection of either loading or maintenance dose on AutoSyp’s user interface, the flow rate was automatically programmed to dispense 60 mL/h or 5 mL/h of 20% MgSO_4_ solution, respectively. During each treatment, the dispensed volume was automatically calculated by the device based on the plunger position and stored on a computer for accuracy analysis of the mean flow rate and total volume delivered. The clinical results for both the loading and maintenance dose administrations were compared to the device’s accuracy during tests performed in the laboratory setting.

**Results:**

Twenty-two women were enrolled in this study. In both the clinical and laboratory settings, the mean flow rate errors for the loading and maintenance dose infusions were under 2%. During 466 h of testing, the device sounded 129 occlusion alarms across 14 subjects. Of these, 71 alarms were false positives.

**Conclusion:**

Results of this study support the use of AutoSyp as a less painful and accurate means of MgSO_4_ administration in clinical environments that lack infusion systems.

There were a large number of false alarms in the current system which will be addressed in future designs. AutoSyp maintains the comfort of intravenous MgSO_4_ administration, but unlike commercially available syringe pumps, it is capable of operating with a variety of syringe brands and sizes and requires no additional consumables. AutoSyp’s appropriate design will benefit its implementation and sustained use in low-resource settings.

**Trial Registration:**

Trial registered prospectively on November 18, 2014 with ClinicalTrials.gov (NCT02296931)

## Background

Severe pre-eclampsia and eclampsia constitute the second leading cause of global maternal mortality, accounting for as many as 50,000 maternal deaths each year [1, 2]. Untreated, pre-eclampsia can lead to stroke, kidney and liver damage, blood clots, pulmonary edema, and the onset of grand mal seizures (eclampsia), endangering the life of both the mother and baby [3].

Magnesium sulfate (MgSO_4_) is an affordable and effective treatment for pre-eclampsia and eclampsia; properly administered, MgSO_4_ reduces the onset of eclamptic seizures by over 50% [3]. Intravenous (IV) MgSO_4_ therapy is typically administered following the Zuspan regimen: an initial loading dose of 4 g MgSO_4_ over 15–20 min followed by a 1–2 g/h maintenance dose, continuing until 24 h after delivery or the last eclamptic seizure. To obtain stable therapeutic plasma concentrations of 2–4 mmol/L MgSO_4_ and avoid MgSO_4_ toxicity that can occur at higher concentrations, infusion pumps are recommended to provide accurate delivery of MgSO_4_ [4, 5].

Ninety percent of deaths worldwide due to pre-eclampsia and eclampsia occur in low-resource settings where infusion pumps are frequently not available [6, 3]. When MgSO_4_ is administered in these settings, the Pritchard regimen is most often followed [7]. In this regimen, 4 g MgSO_4_ is initially delivered via IV and further treatment is delivered via intramuscular (IM) injections. In low-resource settings, the loading dose is often delivered via an IV-push in which clinicians slowly inject the medicine with a syringe over 10 to 20 min [8]. This process is time-consuming and leads to inconsistent flow rates. The IV push is immediately followed by a 10 g MgSO_4_ IM injection, and maintenance doses are delivered via a 5 g IM injection every four hours [9]. Large volume IM injections are painful for patients and the frequent need for injections over the course of MgSO_4_ therapy stresses already overburdened health care personnel in low-resource settings [10].

Most commercially available infusion pumps are priced upwards of $1200 and are not appropriate for use in low-resource settings [11]. The majority require specific consumables that may not be available, do not have sufficient battery life for frequent power outages, and/or have complex user interfaces [12, 13, 6]. To meet this need, we developed AutoSyp, a low-cost, low-power syringe pump. At low production volumes, AutoSyp has a bill of materials cost of approximately $500. The device can work with any syringe and tubing typically found in labour wards. This paper describes results of a pilot study to evaluate the feasibility of using AutoSyp to administer MgSO_4_ intravenously to women suffering from pre-eclampsia at Queen Elizabeth Central Hospital (QECH), the teaching hospital for the University of Malawi College of Medicine, in Blantyre, Malawi.

## Methods

### AutoSyp

The AutoSyp syringe pump was designed to meet the demands and constraints of low-resource environments while maintaining an accuracy and functionality comparable to those of commercially available syringe pumps. A detailed description of the system has been published previously [14]. Briefly, the system incorporates a constant-force spring to drive the syringe plunger forward, allowing the device to operate for up to 66 h before the battery must be recharged. During an infusion, the device continually monitors both the position and back pressure of the syringe plunger using a membrane potentiometer and piezoresistive force sensor, respectively. Safety alarms alert clinicians of flow rate errors exceeding 10% and of occlusions in the cannula or IV line detected by increases in the syringe plunger back pressure. In addition, AutoSyp has a simple user interface, featuring a five-button keypad and a color LCD screen that displays pre-programmed protocols for MgSO_4_ delivery. In previous laboratory testing and clinical testing in a low-resource pediatric ward, AutoSyp demonstrated less than a 3% mean flow rate error across syringe sizes and volumes of 5 to 60 mL and flow rates ranging from 3 to 60 mL/h [14].

### MgSO_4_ delivery protocol

In this study, AutoSyp was programmed to deliver MgSO_4_ following the Zuspan regimen at QECH in Blantyre, Malawi (Table 1). The menu screen for MgSO_4_ delivery allowed users to select between a loading and maintenance dose and select the volume of fluid in the syringe. Given the selection of either loading or maintenance dose, the flow rate was automatically programmed to dispense 60 mL/h or 5 mL/h of 20% MgSO_4_ solution, respectively. During an infusion, the LCD displayed the programmed flow rate, volume dispensed, and the amount of time left until the end of treatment. Becton-Dickinson Luer-Lok Tip syringes (20 ml and 60 ml) were used during benchtop and clinical testing.

**Table 1:** AutoSyp MgSO_4_ Infusion Protocol

	Clinical Dosage	Flow Rate	Volume and Syringe Size
Loading Dose	4 g MgSO_4_ over 20 min	60 mL/h	20 mL in a single 20 mL syringe
Maintenance Dose	1 g/h MgSO_4_ for 24 h	5 mL/h	120 mL in two 60 mL syringes

### Lab evaluation of AutoSyp

In the lab, AutoSyp was set up to deliver the loading and maintenance doses according to the guidelines in IEC 60601-2-24 [15]. The volume of fluid dispensed from the syringe was measured at one second intervals for the duration of the infusion (*n* = 5 for both loading and maintenance dose infusions, Vernier Software & Technology). To assess the accuracy of the plunger position sensor, a computer was connected to AutoSyp to collect data from the sensor at each discrete plunger position throughout the infusion. The dispensed volume was automatically calculated by the device based on the plunger position and compared to the volume of fluid actually dispensed; the mean and maximum of the absolute residual values were calculated for both the loading and maintenance doses.

AutoSyp does not require specific consumables for use. For each syringe brand and size, a scale is used to determine the average shot volume of fluid ejected from the syringe each time the motor ticks. The device software automatically calculates the tick interval given the syringe size, brand, and flow rate input by the user. For this study, the device underwent vigorous calibration and laboratory accuracy testing for a 60 mL B.D. syringe at 5 mL/h and a 20 mL B.D. syringe at 60 mL/h. However, for previous studies, accuracy testing has been performed with 10-, 20-, 30-, and 60- mL syringe sizes at flow rates of 3, 5, 10,40, and 60 mL/h [12].

The accuracy of AutoSyp in delivering a loading and maintenance dose was evaluated in the lab using the volume calculated based on the plunger position. The mean flow rate (*Q*_m_) was determined according to the international standards for non-electrically driven portable infusion devices (ISO 28620) [16].

$$ {Q}_{\mathrm{m}}=\left({0.75}^{\ast }{V}_{\mathrm{N}}\right)/ T, $$

where *T* is the time taken to deliver the nominal volume (*V*_N_).

The percent mean flow rate error was calculated by comparing this mean flow rate to the programmed flow rate of either 60 mL/h or 5 mL/h. The total volume dispensed was compared to the programmed delivery volume at the end of the run.

### Clinical protocol

The accuracy of AutoSyp was evaluated for MgSO_4_ delivery in pre-eclamptic women. All procedures were approved by the Rice University Institutional Review Board (IRB #14-081F) and the National Health Sciences Research Committee in Malawi (Protocol #1312).

All subjects gave written informed consent after reviewing the study procedures with a nurse. Inclusion criteria required that all subjects be at least 18 years of age, be pregnant or up to 24 h postpartum, have a systolic blood pressure of at least 140 mmHg or a diastolic blood pressure of at least 100 mmHg, be diagnosed with pre-eclampsia, and be deemed to benefit from treatment with MgSO_4_ by healthcare providers. Patients were ineligible for enrollment if they were diagnosed with eclampsia, had experienced convulsions by the time of enrollment, or had received MgSO_4_ therapy within the preceding 24 h.

Upon enrollment, subjects received IV MgSO_4_ therapy with AutoSyp. Any other treatments prescribed by the patients’ doctors, which included 0.9% saline, Ringer’s Lactate Solution, Oxytocin, and Hydralazine, were given through a separate IV line.

Subjects were monitored by study nurses and technicians at least every 5 min during the loading dose and every 30 min during the 24 h maintenance dose. During this time, the subject’s blood pressure was recorded and the subject was assessed for symptoms of MgSO_4_ toxicity. If at any point during an infusion the clinical or research staff felt that the use of AutoSyp was inhibiting delivery of MgSO_4_, such as if there were frequent occlusion or device accuracy alarms, AutoSyp was stopped and the subject continued MgSO_4_ therapy using the standard method of care.

### Clinical evaluation of AutoSyp

Before the start of the trial, maternity ward nurses received training on the study protocol and device usage. Throughout the course of the study, a trained nurse was always present to (1) identify patients in need of MgSO4; (2) insert the cannula; (3) prepare the syringe and tubing with 20% MgSO_4;_ (4) program the device to administer the proper infusion; (5) respond to occlusion alarms; (6) identify any potential complications of treatment; and (7) follow up on the patient’s care. Study personnel were present at all times during device use and supervised all interactions with the device. All clinical decisions were made by hospital staff.

**Fig. 1:**
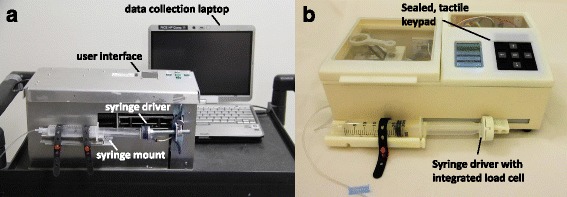
The AutoSyp Device. **a** AutoSyp can be used with all major brands of syringes and giving sets. The simple user interface includes an LCD screen and keypad. **b** The redesign includes a simplified drive mechanism used to rewind the spring. A robust load cell is used in place of the pressure pad for occlusion detection

During clinical use, a laptop computer was connected to AutoSyp to collect and store the syringe plunger position and back pressure from the device’s sensors (Fig. 1a). Data were sampled at each discrete plunger position. The device used the plunger position values to calculate the volume dispensed throughout the infusion. An infusion is defined as every time AutoSyp was programmed to dispense fluid from a new syringe; therefore, one maintenance dose administration consisted of two 60 mL infusions. Errors in the mean flow rate and the total volume dispensed were evaluated for each infusion using the methods described above. During testing, the volume in the syringe was also manually monitored by the technician every 30 min, so that in the case of loss data or a device malfunction, clinical staff would be made aware of any large errors in drug delivery.

At every occlusion alarm, nurses and technicians took notes on the status of the patient’s tubing and cannula and flushed the cannula before continuing treatment. Data were analyzed to determine the number of true occlusions detected and the number of false occlusion alarms. An occlusion alarm was deemed true if the nurse or technician found the tubing to be kinked or unopened or if flushing the patient’s cannula resulted in a return to normal infusion pressures and fluid flow. An occlusion alarm was deemed false if tubing and cannula appeared to be clear and if fluid flow from the syringe was not in any way impeded.

## Results

### Benchtop accuracy results

The mean residual difference between the volume of fluid actually dispensed by AutoSyp and that calculated based on the syringe plunger position was 0.10 mL for the 20-mL loading dose and 0.40 mL for the 60-mL maintenance dose. The maximum residual was 0.34 mL for the loading dose and 1.34 mL for the maintenance dose. Because the syringe plunger position could be used to calculate dispensed volume with high accuracy, it was used in all further assessments of the accuracy of AutoSyp. Table 2 lists benchtop accuracy for both the loading and maintenance doses. The mean errors for both the volume dispensed and flow rate were less than 2%.

**Table 2:** Accuracy of MgSO_4_ Delivery

		Laboratory Performance		Clinical Performance	
		Mean Error	95% CI	Mean Error	95% CI
Loading Dose	Total Volume Dispensed	−1.16%	[−1.28, −1.04]	−1.48%	[−1.72, −1.25]
	Flow Rate	−1.83%	[−2.13, −1.53]	−1.75%	[−2.06, −1.46]
Maintenance Dose	Total Volume Dispensed	−1.43%	[−1.89, −0.97]	−1.13%	[1.30, −0.97]
	Flow Rate	−1.67%	[−1.92, −1.42]	−1.11%	[−1.22, −0.10]

### Clinical accrual

The study was performed between July and October 2015. All patients eligible for the study were presented with the option to participate. Informed consent was obtained from 22 eligible patients. Table 3 summarizes the demographic information for study participants. Of the 22 patients, two were postpartum and 20 gave birth during the 24 h of treatment. For those brought to the operation theater for caesarean delivery, the treatment was paused for the extent of the operation and then continued immediately afterward. There were no convulsions or maternal deaths among the study participants.

**Table 3:** Patient Demographics

Number of subjects	22
Patient Demographics	Median (Range)
Age (years)	26 (19–37)
Gestational Age (weeks)	36 (28–39)
Antenatal Care Visits	3 (1–5)
Gravidity	2 (1–5)
Systolic Blood Pressure (mmHg)	160 (139–230)
Diastolic Blood Pressure (mmHg)	104 (81–133)
Proteinuria	+3 (+1 − +4)

Seventeen of the 22 subjects enrolled in the study completed 24 h of MgSO_4_ treatment using AutoSyp. AutoSyp successfully delivered 21 of 22 intended loading dose infusions and 35 of 39 intended maintenance dose infusions. Reasons for a subject’s discontinuation of MgSO_4_ therapy on AutoSyp included: frequent occlusion alarms caused by a faulty occlusion sensor (1), a device accuracy alarm due to an undetected occlusion (1), a device accuracy alarm due to the wrong syringe size and volume being entered by the clinician (2), and an unexpected shortage of MgSO_4_ (1). Each of these patients completed MgSO_4_ therapy with the Pritchard regime, the QECH standard of care, and experienced no adverse side effects.

### Clinical accuracy results and occlusion alarms

During the clinical trial, the continuous data collection system occasionally malfunctioned, preventing data analysis for four of the 21 completed loading dose infusions and eight of the 35 completed maintenance dose infusions. Problems encountered with the data collection system include: faulty performance of the position sensor related to damage sustained during transport to Malawi (9); clinicians frequently needing to pause and restart the device due to frequent occlusion alarms (2); and data collection at the end of an infusion not being properly saved by the technician (1).

Table 4 provides a list of attempted, completed, and analyzed loading and maintenance dose infusions. In all, accuracy data were analyzed for 17 loading dose and 27 maintenance dose infusions. The results of the clinical accuracy analysis, including the means and ranges of the flow rate and volume dispensed errors, are presented in Table 2. All mean errors were less than 2%.

**Table 4:** Infusion and Data Collection

Loading Dose Infusions	
Attempted	22
Completed	21
Analyzed	17
Maintenance Dose Infusions	
Attempted	38
Completed Delivery	35
Analyzed	27

All recorded occlusion alarms from the 60 attempted infusions were reviewed. For eight of the 22 subjects, no occlusion alarms were recorded. A total of 129 recorded occlusion alarms occurred over 466 h of total testing during infusions for 14 of the 22 test subjects. Among the 129 alarms, 71 were false positives, one was a false negative, 39 were true positives, and 19 could not be categorized due to a possible connection issue with the force sensor. Of the true positives, only one alarm was due to an occluded cannula while the rest were caused by kinked or unopened tubing.

## Discussion

AutoSyp was designed to meet the needs and constraints of low-resource hospitals, and has previously been proven to administer fluids within a 3% mean flow rate error in healthy adult and infant populations. In this study in a Malawian referral hospital, AutoSyp delivered MgSO_4_ treatment in accordance with the Zuspan regimen to 22 pre-eclamptic women, achieving a mean flow rate error of −1.83% and −1.67% for the loading and maintenance dose infusions, respectively. No subjects experienced an eclamptic seizure during their enrollment in this study. The device provided up to 12 h of continuous fluid administration before a clinician was audibly and visibly alerted to refill the syringe, and throughout the entire course of treatment the LCD screen displayed the actual volume of fluid administered and the time left in the infusion.

Fourteen of the 22 subjects experienced at least one occlusion alarm; while 71 of the total 129 alarms were false positives, only one false negative occurred throughout the entire 466 h of testing. Because of the large number of false alarms for occlusions generated during this study, AutoSyp was modified to include a more reliable occlusion detection system through the incorporation of a compression load cell into the device’s plunger driver. The system has been calibrated to correspond to occlusion thresholds ranging from 100 to 700 mmHg, which in future studies will reduce the number of false alarms. There were also a number of true positive alarms for occlusions during this study. In the future, better training on AutoSyp use may be able to address setup issues leading to kinked or unopened tubing.

While promising, this study to evaluate AutoSyp has several limitations. We did not directly compare the performance of AutoSyp to the current standard of care, and so evaluations in patient outcome and protocol feasibility between the two methods could not be performed. However, in future studies, patient outcomes, patient comfort, and clinician feedback will be assessed for comparison between methods. In addition, all subjects were frequently monitored by specified study technicians and nurses; further studies are needed to characterize the performance of the AutoSyp in understaffed hospitals. Further studies will also address the high rates of data collection errors (12/56 infusions) by automatically recording dispensed volumes onto an SD card internal to the device. This will greatly simplify data collection and allow the device to be set up according to standard clinical practice.

Despite these limitations, results of this study support the use of AutoSyp as a less painful and more accurate means of MgSO_4_ administration in clinical environments that lack infusion systems. While the Pritchard and Zuspan regimens are both appropriate methods to treat severe pre-eclampsia, continuous IM injections are time consuming for clinical staff and cause significant pain and possible abscess formation [7]. The problem is complicated when only 20% MgSO_4_ solution is available, in which case, delivery of a single 5 g MgSO_4_ dose requires that 25 mL be injected into the buttock.

Administering MgSO_4_ intravenously to reduce patient discomfort in low-resource settings is supported in the study of the Springfusor IV pump, a completely mechanical pump which straps to the patient’s arm to deliver MgSO_4_ therapy; over 95% percent of subjects with Springfusor reported pain levels associated with MgSO_4_ administration as acceptable or very acceptable [10]. However, Springfusor has a per-use consumable cost of $4.40–$9.40 for flow-controlled tubing and is only compatible with 10 mL and 30 mL Braun Syringes [17, 18]. During this study, B-D syringes and tubing were used to connect AutoSyp to the patient. These syringes and tubing cost approximately $4.80/patient when purchased in small quantities. However, in future usage, AutoSyp will be able to maintain the comfort of IV MgSO_4_ administration while being capable of operating with whichever syringe brands and sizes are available on the ward.

Figure 1b shows a photograph of the functionally refined AutoSyp. Further studies will also record the frequency of AutoSyp’s use as a means of MgSO_4_ delivery and patient outcomes in comparison to the standard IM regimen.

## Conclusions

Given the lack of appropriate low-cost infusion systems, low-resource settings often rely on time-consuming, painful, and less-accurate IV push and IM injections to administer MgSO_4_ to pre-eclamptic and eclamptic women. AutoSyP offers a practical alternative for IV MgSO_4_ administration in these settings. In a Malawian referral hospital, AutoSyp administered MgSO_4_ to pre-eclamptic women with less than a 2% mean flow rate error for both the loading and maintenance dose infusions.

## Abbreviations

IM: Intramuscular
IV: Intravenous
MgSO_4_: Magnesium Sulfate
QECH: Queen Elizabeth Central Hospital
